# Estimating Genetic Ancestry Proportions from Faces

**DOI:** 10.1371/journal.pone.0004460

**Published:** 2009-02-18

**Authors:** Yann C. Klimentidis, Mark D. Shriver

**Affiliations:** 1 Department of Anthropology, University of New Mexico, Albuquerque, New Mexico, United States of America; 2 Department of Anthropology, Pennsylvania State University, University Park, Pennsylvania, United States of America; University of Utah, United States of America

## Abstract

Ethnicity can be a means by which people identify themselves and others. This type of identification mediates many kinds of social interactions and may reflect adaptations to a long history of group living in humans. Recent admixture in the US between groups from different continents, and the historically strong emphasis on phenotypic differences between members of these groups, presents an opportunity to examine the degree of concordance between estimates of group membership based on genetic markers and on visually-based estimates of facial features. We first measured the degree of Native American, European, African and East Asian genetic admixture in a sample of 14 self-identified Hispanic individuals, chosen to cover a broad range of Native American and European genetic admixture proportions. We showed frontal and side-view photographs of the 14 individuals to 241 subjects living in New Mexico, and asked them to estimate the degree of NA admixture for each individual. We assess the overall concordance for each observer based on an aggregated measure of the difference between the observer and the genetic estimates. We find that observers reach a significantly higher degree of concordance than expected by chance, and that the degree of concordance as well as the direction of the discrepancy in estimates differs based on the ethnicity of the observer, but not on the observers' age or sex. This study highlights the potentially high degree of discordance between physical appearance and genetic measures of ethnicity, as well as how perceptions of ethnic affiliation are context-specific. We compare our findings to those of previous studies and discuss their implications.

## Introduction

The feeding ecology of humans demands complex social behavior and extensive cooperation [1], resulting in multilevel selection [2]–[4] for strong within-group cohesion and out-group circumspection [5]–[7]. Only under evolutionarily ‘recent’ conditions do humans routinely encounter individuals who differ substantially in physical appearance. It is therefore doubtful that any cognitive mechanism evolved to explicitly deal with perceiving physical characteristics associated with different groups [8]. Kurzban et al. [9] have argued that in today's society, the tendency to categorize individuals by race is not inevitable, and only exists in as much as it encodes information about social alliances, thus highlighting how race is socially perceived in a highly context-specific way. On the other hand, Gil-White [10] argues that our cognitive architecture has evolved in such a way as to “essentialize” ethnic groups as if they were different species, and is initially determined by assessment of morphological characters, then reinforced or overridden by knowledge of common descent. However, he finds that among Mongols and Kazakhs, facial characters were of limited reliability in assigning a pictured individual to an ethnic group [11]. Nonetheless, it is interesting to note that the subjects are reported as initially feeling very confident about being able to detect ethnicity from facial features [11].

Phylogenetic evidence suggests an ability in many organisms to discriminate between individuals based on relatedness [12], including chimpanzees [13], and humans [14], through a recognition heuristic [15], and/or through self-referent phenotype matching [16]. Discriminating based on facial appearance may provide a selective advantage in mating [14], [17], and cooperative interactions [18], [19] since it can reveal cues about relatedness and shared group membership. Ethnographic evidence shows that relatedness and kinship are important features of human sociality, and that over human evolutionary history, cognitive and cultural mechanisms may have been selected to associate group membership based on these criteria [10]. Across societies, kinship terms are culturally co-opted and extended to a wider set of non-kin within the family, tribe, or ethnic group [20]–[22]. This way of thinking about kinship was likely adaptive at the group and individual levels in ensuring reciprocity and solidarity within groups by extending emotions associated with the closest of kin to all members of the group. The evolutionary mechanisms associated with distinguishing one's groups vs. other are likely in operation when humans are faced with the novel environment of today's societies that are often characterized by the presence of other individuals who are phenotypically different. Jones (2000) has suggested that today we see the world as being divided into large, geographically defined kin groups defined by “underlying natural commonalities.” Indeed, even in today's large nation-states, we find leaders playing to their public's emotions by evoking a feeling of kinship among citizens (Johnson et al. 1987; Salmon 1998).

There has been extensive research on facial recognition and how it differs according to the race of the observer and the race of the observed. Studies consistently show an own-race effect in which individuals are better at recognizing faces from their own racial group than faces from other racial groups, and the magnitude of this effect is generally attributed to the level of experience and exposure that individuals have to faces of different races [23], [24]. There have been several studies that examine the discrepancy between self-identified race and observed race. Harris and Sim [25] examined the relationship between self-reported race and race as perceived by one observer. They find a high degree of concordance, except for faces of American Indian and multiracial individuals. Harris [26] examined the variation in how individuals identify a person's race, and finds that self-reported race is more likely to be confirmed for individuals who belong to a single racial group, especially Whites and Blacks. Match rates for multiracial and Latino faces are the lowest. Harris also finds that observers who had more experience with people of other races were better at categorizing the ethnicity of individuals. Habyarimana et al. [27] find that among a sample of US university students, observers are unable to correctly identify the ethnicity of photographed individuals more than 30 percent of the time, that observers more often correctly categorize co-ethnics, and that Latinos are less successfully categorized than White, Asians, or African Americans.

According to Condit et al. [28], it is likely that there is a common social perception that racial groupings correspond to differences in physical appearance, that physical appearance is caused by genetics, and “therefore that race has a genetic basis”. Parra et al. [29] were the first to examine the relationship between genetic admixture and estimation of “Color” from facial appearance and skin color. This estimate of “Color” was based on the evaluations of a sample of Brazilians by two health care workers who examined skin pigmentation on the arm, hair color and texture, and the shape of the nose and lips. The relationship between the resulting estimated “Color” variable and African genetic admixture, based on 10 Ancestry Informative Markers (AIMs), was examined. They find a high degree of overlap in the levels of African genetic admixture between the Brazilians who were classified as Black, Intermediate, and White, but a much smaller degree of overlap between Brazilians and the putative parental un-admixed populations from Portugal and the island of Sao Tome, off the coast of West Africa. They conclude that “Color” is a relatively poor predictor of degree of African genetic admixture. More recently, using a panel of 40 AIMs, Suarez-Kurtz et al. [30] examined the relationship between self-identified “color” categories and proportion of African genetic admixture. Although they find significant overlap in African admixture between the three categories (Whites, Intermediates, Blacks), the three groups differed significantly from each other.

Hispanics are a biologically and culturally heterogeneous group produced by 400 years of mixture between Native Americans and people of European and African ancestry. Genetic evidence has shown that Hispanics have wide ranges of Native American, European and African admixture proportions and vary with respect to phenotypes such as skin color [31]–[33]. It is also likely that biological and cultural heterogeneity have produced variation in facial features in this group [34], [35], in addition to variation in skin color [36]. This variation in phenotypes may affect how individuals identify and perceive themselves and others [5], [37], [38].

In this study, we assess the degree of concordance between observer-estimated and genetic-estimated Native American admixture by showing a series of 14 photographed self-identified Hispanic/Latino individuals to a sample of New Mexicans recruited at an Albuquerque Motor Vehicle Division (MVD) waiting area, and at a local university. We also examine how characteristics of observers, such as age, sex, ethnicity, socio-economic status (SES), and community origin vary with the measure of concordance.

## Methods

### Facial Photographs

From a sample of 55 self-identified Hispanics/Latino Americans who were students at Pennsylvania State University, we chose fourteen individuals (8 males and 6 females) between the ages of 18 and 33 whose family origins were in Europe and the Americas. The 14 were chosen because they had low African (10% or less) and East Asian (9% or less) genetic admixture, and because they had a wide range of Native American ancestry. The facial photographs were cropped so as to show as little clothing as possible. Raters were shown two 7×8 cm photos, side by-side: one frontal and one profile. The same 14 individuals were shown to all raters, in the same order. Subjects in the photos ranged in Native American genetic admixture from 0 to 63% (actual values: 0, 0, 5, 13, 16, 17, 21, 25, 35, 47, 48, 50, 62, 63). The faces were shown in random order with respect to these ancestry proportions. All subjects gave written informed consent and the study was approved by both the University of New Mexico and the Pennsylvania State University Institutional Review Boards.

### Genetic Admixture measurements

DNA was obtained from all subjects in the photos, and it was typed for 176 AIMs by DNAPrint Inc. (Sarasota, FL.) [39]. Individual estimates of genetic admixture were obtained using the maximum likelihood estimation method first described by Hanis et al. (1986), and implemented in the IAE (Individual Admixture Estimation) program developed by Mark Shriver and Carrie Pfaff. These estimates rely on allele frequencies of AIMs in four putative un-admixed parental populations [39]. Since we had 4 individuals whose family origins were in South America, and the rest whose family origins were in North/Central America, we performed the analyses with all 14 individuals, and then with the these four individuals removed. It should be noted that the frequencies of the utilized AIMs have been shown to not differ significantly across current-day populations of the Americas [32], [40].

### Raters

A total of 241 subjects (see Table 1) were recruited to fill out a questionnaire and give their estimates of admixture for the 14 facial photos. 134 subjects were University of New Mexico students recruited from introductory Biology and Anthropology classes. 107 subjects were recruited at a Bernalillo County (Albuquerque, New Mexico) Motor Vehicle Division (MVD) waiting room. Anyone over the age of 18 who could read English was eligible to participate. Observers were given the option to circle a number between 0% and 65% NA genetic admixture, shown in increments of 5%, for each of the fourteen photographed individuals. Observers were also asked about their own age, ethnicity, self-estimated African, East Asian, European, and Native American ancestry proportions, where they lived for most of their life, income, and education (only for MVD observers).

**Table 1:** Sample Characteristics.

		Mean Age	Hispanic	Native American	White	African American	Asian
UNM students (n = 134)	Male = 62	21.6	42	8	61	3	5
	Female = 72						
MVD (n = 107)	Male = 58	36.8	48	14	32	5	2
	Female = 49						

### Statistical analyses

In order to assign an error score for each subject, we computed the average Euclidean distance between the observer and the genetic estimate of admixture, over all photographs. We used the following formula to determine this distance:
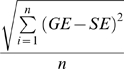
GE is the genetic estimate, SE is the subject's estimate, and n is the number of faces for which the subject gave an estimate, since some subjects did not give an estimate for every photo. To determine what the distance would be if someone had estimated randomly, we simulated 241 such individuals and computed the average distance/error score. T-tests are used to determine if there is a significant difference between the mean error scores of the observers and the mean error score of the simulated random observers, as well as to determine whether there is a difference between the mean error score of the MVD and student sample. We used Kruskal-Wallis and Mann-Whitney tests to determine if there are differences in average error score (i.e. concordance) according to the ethnic groups of the observers. To further examine variation in error score, we use multiple linear regression to examine the relationship between rater error score and the rater's age, self-estimated Native American and European ancestry proportions, education level, and income. To determine whether there are differences in error score according to where subjects lived for most of their life, we coded subjects according to whether they lived most of their life in the southwestern United States or not. We then used a Mann-Whitney test to test for a difference in the mean error score between these two groups. To determine whether subjects consistently over- or under-estimated the degree of NA admixture, we averaged the differences between the estimated and genetic estimates over all photos, for each subject. Statistical analyses were performed in SPSS version 12.0.

## Results

### Observer vs. genetic estimated admixture

**Figure 1:**
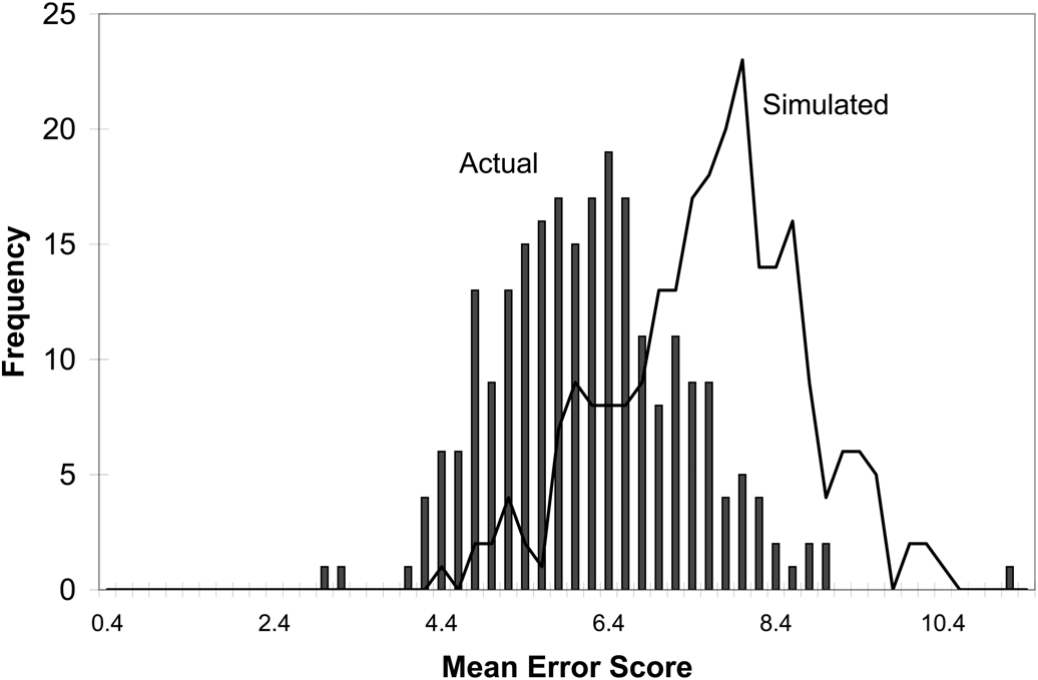
Distribution of mean error scores for actual raters (histogram blocks) and for simulated random raters (line). The best possible error score is 0.36.

The mean error score for all 241 observers is 6.074±1.141. The mean error score of 241 simulated observers who assigned admixture estimates randomly is 7.487±1.134 (see Figure 1). We find a highly significant difference between these two means [t-test: t_(478)_ = −13.618, p = 1.51×10^−36^], indicating that, on average, observers are able to estimate admixture levels from photographs better than chance. Approximately 89% of observers are able to estimate better than the average simulated observer who estimates randomly. The error score for a simulated observer who estimates as close as possible to the genetic estimates is 0.36. This score is slightly higher than 0 due to the fact that observers can only estimate in increments of 5% admixture. The observers' performance is therefore much closer to being random than it is to being perfect: 6.07 (observers) vs. 7.49 (simulated random observers) vs. 0.36 (simulated perfect observer). The non-response rate is 1.7% (57 out of 3374 possible responses). This could lead to a bias, albeit small, if subjects avoided guessing the ancestry of individuals that they were unsure of. However, none of the faces had a disproportionately large number of missing estimates. The number of missing estimates per face varied between 2 and 8, out of 241 possible estimates.

The mean error score for university students (5.891) is significantly lower than it is for subjects recruited at the MVD (6.302) (t-test: t_(237)_ = 2.803, p = 0.005), meaning that the concordance between genetic and observer estimates of admixture is higher among university students. This difference may be responsible for the positive and significant relationship (β = .023, p<0.001, r^2^ = 0.055) between age and error score seen in the entire sample. We find no difference in mean error score between males (6.005) and females (6.141) [t-test: t_(237)_ = −0.923, p = 0.357].

When we repeat the analyses without the photos of the four individuals who have South American family origins, the mean error score for all 241 observers is 7.17±1.78 and 9.55±1.6 for the simulated random estimators [t-test: t_(478)_ = −15.552, p = 9.79×10^−44^]. We find no significant difference between the student (7.054) and MVD sample (7.311) [t-test: t_(237)_ = 1.109, p = 0.27], a significant relationship between age and error score [β = .023, p = 0.022, r^2^ = 0.022], and a significant difference between males (6.933) and females (7.398) [t-test: t_(237)_ = −2.032, p = 0.043].

We also find no significant difference in average distance score between individuals who reported living most of their life in the Southwest US (n = 197) and those who didn't (n = 23) [z = −0.19, p = 0.85]. We find a similar result [z = −0.94, p = 0.35] when we repeat the analyses without the photos of the four individuals who have South American family origins.

### Ethnicity and observer-genetic concordance

**Figure 2:**
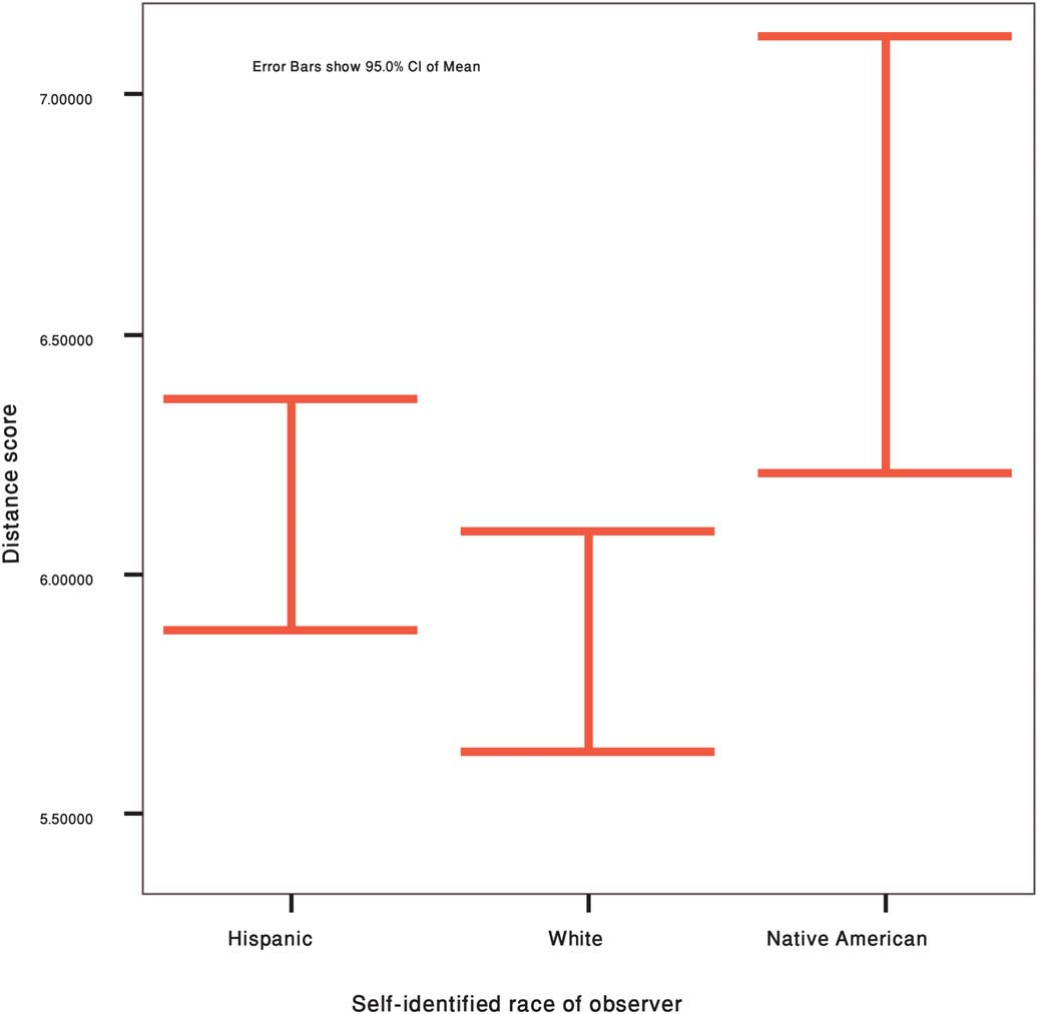
Average Euclidean distance score by race/ethnicity. Error bars show 95% CI of Mean.

We compared the average error score according to the self-identified ethnicity/race of the observer, and find that there are significant differences in the average error scores between groups (see Figure 2). The Kruskal-Wallis test for differences in the mean distance scores between Hispanics, Whites and Native Americans is significant [Χ^2^ = 10.383, df = 2, p = 0.006]. The Mann-Whitney test shows that Native Americans have, on average, a higher mean error score than both Whites [Z = −3.082, p = 0.002], and Hispanics [Z = −2.179, p = 0.029]. Further confirming this finding, linear regression between the error score and self-estimated ancestry proportions shows a positive relationship with Native American ancestry [β = 0.010; r^2^ = 0.082; p<0.001], and a negative relationship with European ancestry [β = −0.009; r^2^ = 0.092; p<0.001].

When we repeat the analyses without the photos of the four individuals who have South American family origins, the test for differences in the mean distance scores between Hispanics, Whites and Native Americans is not significant [Χ^2^ = 0.880, df = 2, p = 0.644], and none of the pair-wise differences are significant. However, the regression between the error score and self-estimated ancestry proportions shows a positive relationship for Native American ancestry [β = 0.008; r^2^ = 0.023; p = 0.025], and a negative relationship for European ancestry [β = −0.009; r^2^ = 0.042; p = 0.002].

### Multiple regression of predictor variables of distance score

According to the multiple regression model, none of the independent variables are consistently statistically significant predictors of the error score across each of the separate samples and the combined sample (see Table 2). Among the UNM student sample, there are two variables that are significant: age [β = 0.062, p = 0.006] and self-estimated EU admixture proportion [β = −0.008, p = 0.028]. Self-estimated NA and EU admixture are negatively correlated with each other [r^2^ = 0.51, p<0.001]. Among the MVD sample, none of the independent variables are statistically significant predictors of the error score. Among the entire sample, age is the only significant predictor of the error score [β = 0.014, p = 0.036], but this is driven by the highly significant association in the student sample.

**Table 2:** Multiple regression with error score as the outcome variable, for each sample separately, and for both samples together.

	UNM sample		MVD sample		Both samples	
	Beta	p-value	Beta	p-value	Beta	p-value
Age	0.062	0.006	0.006	0.624	0.014	0.036
Income	0.000	0.821	0.000	0.275	0.000	0.702
Education			−0.042	0.561		
Self-Estimated NA admixture	0.001	0.893	0.010	0.060	0.006	0.101
Self-Estimated EU admixture	−0.008	0.028	−0.002	0.624	−0.005	0.080

When we repeat the analyses without the photos of the four individuals who have South American family origins, there are no significant relationships between the independent variables and the error score, except for age in the student sample (p = 0.008).

### Direction of the discrepancy

**Figure 3:**
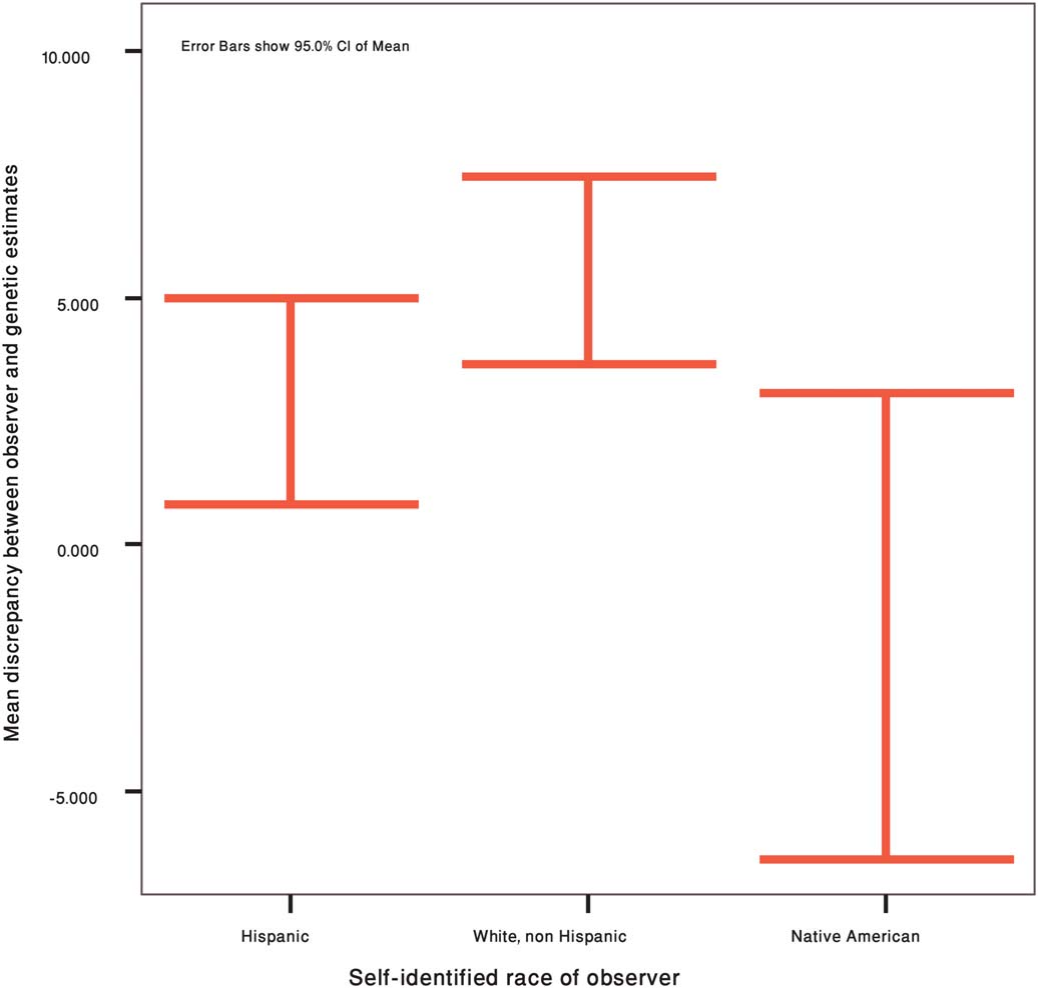
Averaged difference between observer and genetic estimates by self-identified race of observer. Positive values indicate an overall overestimation of NA ancestry by the observers, compared to the genetic estimates. Error bars show 95% CI of Mean.

The direction of the discrepancy between the genetic and observer estimates is positive if observers, on average, overestimated the degree of NA admixture, and negative if they underestimated the degree of NA admixture. The average discrepancy is 4.08%±9.63, meaning that observers overestimate NA admixture, on average, by about 4.1%. According to t-tests, the mean discrepancy does not differ by sample (p = 0.111) or by sex (p = 0.083). Using the Kruskal-Wallis test we find a significant difference between observer race (Hispanics, Native Americans, Whites) [X^2^ = 10.175, df = 2, p = 0.006] (see Figure 3). Whites and Hispanics tend to overestimate the degree of NA admixture (by 5.56% and 2.91%, respectively), over all photographs, while Native Americans tend to underestimate (by 1.65%). The Mann Whitney test shows that the difference between Whites and Native Americans is statistically significant [Z = −3.02; p = .003].

When we repeat the analyses without the photos of the four individuals who have South American family origins, the average discrepancy is 7.93%±9.74. The average discrepancy does not differ by sample (p = 0.194) or by sex (p = 0.172). We also find a similar pattern of differences across observer race [X^2^ = 11.413, df = 2, p = 0.003], and between Native American and White observers [1.20% vs. 9.31%, respectively; Z = −3.15 p = 0.002]

## Discussion

This study shows that the degree of concordance between genetic and observer estimated Native American ancestry proportions for individuals in this sample of New Mexicans is slightly but significantly higher than if observers were to estimate randomly. The error in estimation by the observers (6.07) is much closer to random estimation (7.49) than it is to perfect estimation (0.36), suggesting either that facial features are not perfectly reliable indicators of ancestry as was shown in Brazil [29], [30], or that individuals are not very closely attuned to the phenotypic cues of group differences. We discuss our findings, relate them to previous findings, and discuss their implications regarding human social behavior.

We find it difficult to establish that the degree of concordance varies with age. Younger individuals show a higher degree of concordance (lower error) for the entire sample. This relationship is significant only in the student sample and not in the MVD sample. Since the students perform better than the subjects at the MVD, the effect of age may actually be an effect of being a college student (education, SES, etc…). Alternatively, this age effect may be due to the age of the photographed individuals who are between the ages of 18 and 33. Perhaps, mating and social considerations influence the amount of attention paid to cues of group affiliation, and the payoffs to the detection of these cues is highest at the ages when mating effort is highest, and in this case, when both the observer and the persons in the photograph are of the same age.

We also find that the degree of concordance varies according to self-identified ethnicity and self-assessed ancestry proportions of the observers. Most notably, those who self-identify as White, and those who report having a higher degree of European admixture have a higher degree of concordance between their estimates and the genetic estimates (i.e. lower error score) than observers who identify as Native American and report having lower levels of European ancestry. Since most of the photographed individuals were closer to having 0% Native American ancestry than 100%, these results are consistent with the results of Habyarimana et al. [27] who find that the rate of correct ethnic identification of in-group members is higher than for out-group individuals. They may also be consistent with the contact/differential experience hypothesis for facial recognition which proposes that facial recognition success is higher for in-group members than for out-group members because of having more contact/experience with members of one's in-group [41]. It could be that Native Americans in this sample have less contact/ experience with individuals who have low levels of Native American ancestry than Whites do. It may therefore be less socially important for them to observe variation in other-race individuals as much as it would be in own-race individuals. Although this hypothesis applies to facial recognition of own-race versus other-race individuals, it can nonetheless shed some light on these findings.

These findings have several implications. In the medical field, race may sometimes play an important role in how disease risk is assessed, and accurate records of ethnicity are important to accurately understand population differences with respect to health-related phenotypes. According to several reports, the agreement between self-reported ethnicity and administration records that are based on visual inspection by medical staff is tenuous, especially for Hispanics and Native Americans [42]–[46]. The results presented here confirm this tenuous relationship. For these reasons, it has been argued that self-reported ethnicity is preferable to medical record data. However, self-reported data may provide an incomplete picture of ethnicity, especially in admixed populations [47]–[49]. For example, in one study of Puerto Rican women living in New York City, subjects have anywhere between 0 and 90% Native American ancestry and 0 to 60% African ancestry [32], and among several samples of African Americans, subjects show anywhere between 0 and 80% European ancestry [50]–[52].

These results also have implications when considering the history of admixture, sexual selection, and the genetics of complex traits. For example, it may be that after several generations of sexual selection for facial appearance, the genetic variants that are responsible for those traits would no longer be in linkage disequilibrium with other population-specific genetic variants [29]. This process would result in dissociation between these traits and the estimate of genetic admixture for the rest of the genome. Depending on their specific history, populations differ in how they are stratified with respect to admixture. This will affect the strength of the correlation between genetic admixture estimated from genetic markers scattered throughout the genome and any phenotypes that differ between parental populations. Factors such as a long time since initial admixture, non-continuous gene flow, and assortative mating will decrease the degree of admixture stratification, and hence the relationship between overall admixture and phenotypes that are different between parental populations and that are controlled by just a few loci [53].

The results from this paper demonstrate that there is a relationship between social and biological measures of race/ethnicity but that it is far from perfect and is context specific. It should be noted that we are testing for this relationship at a very high resolution (within an ethnic group), suggesting that at broader levels of race/ethnicity groupings, the relationship would likely be closer. The results also suggest that the degree to which humans are attuned to cues of group membership extends to cues of kinship as assessed by physical appearance, in addition to other cues such as language and cultural markers.

### Conclusions

Our findings warrant further research in other admixed populations such as African Americans, as well as more studies that can isolate specific facial phenotypes and how they vary with admixture. It is also important to determine whether experience from training with photos and corresponding genetic admixture measures would increase the ability of individuals to gauge ancestry/admixture from facial features.
